# Group A streptococcus endocarditis in children: 2 cases and a review of the literature

**DOI:** 10.1186/s12879-019-3736-6

**Published:** 2019-01-31

**Authors:** Nao Ogura, Kouki Tomari, Tomotada Takayama, Naoya Tonegawa, Teppei Okawa, Takashi Matsuoka, Mami Nakayashiro, Tsutomu Matsumora

**Affiliations:** 1 Okinawa Prefectural Nanbu Medical Center & Children’s Medical Center, Department of General Pediatrics, Okinawa, Japan; 2 Okinawa Prefectural Nanbu Medical Center & Children’s Medical Center, Department of Pediatric Cardiology, Okinawa, Japan

**Keywords:** Group A *Streptococcus*, Infective endocarditis, Serotype, *emm* type

## Abstract

**Background:**

Infective endocarditis (IE) is defined as endocarditis caused by microorganisms (bacteria or fungi) involving either the heart or great vessels. The clinical course of IE can be complicated by cardiac dysfunction and bacterial embolization to virtually any organ. *Staphylococcus aureus* and viridans group streptococci are the most common causative organisms, whereas group A *Streptococcus* (GAS) is less common. Although some GAS serotypes have been associated with severe disease, there are few reports of IE associated with GAS serotypes. Here, we report two cases of GAS endocarditis and review the associated literature.

**Case presentations:**

Patient 1 was a previously healthy 14-year-old girl who developed bacteremia and disseminated intravascular coagulation secondary to left foot cellulitis. She was administered intravenous antibiotics. Two of three blood cultures grew *Streptococcus pyogenes* (T6 M6, *emm*6.104). Three days later, a new systolic ejection murmur was heard and echocardiography showed mitral regurgitation with mitral valve vegetation. Because of the resultant severity of the mitral regurgitation, she underwent mitral valve repair after 10 weeks of antibiotic treatment. Patient 2 was a 17-month old boy who presented with a fever. He had a history of spontaneous closure of a ventricular septal defect (VSD). He was started on intravenous antibiotics for possible bacteremia. Two consecutive blood cultures with an interval of more than 12 h grew *S. pyogenes* (T4 M4, *emm*4.0). Five days later, echocardiography showed vegetation on a membranous ventricular septal aneurysm. The patient responded well to antibiotics, and recovered fully with no complications.

**Conclusions:**

Although both patients developed GAS endocarditis, patient 1 did not have any predisposing conditions for IE, and patient 2 had a only a low-risk predisposing condition, a VSD that had closed spontaneously at five months of age. We found twelve reports in the literature of GAS endocarditis with information on serotypes. All patients in these reports had GAS endocarditis caused by serotypes generally associated with milder infections, but no specific risk trends were identified. A greater accumulation of cases is necessary to more clearly elucidate the association between GAS IE and specific serotypes.

## Background

Infective endocarditis (IE) is defined as endocarditis caused by microorganisms (bacteria or fungi) involving either the heart or great vessels. The course of IE can be complicated by embolization to virtually any organ, depending on whether the disease involves the right or left side of the heart. The mortality rate in children is 5–10% [1–3]. Although reports vary, IE occurs less commonly in children, accounting for between 0.05–0.12 in every 1000 to approximately 1 in every 1300–2000 pediatric admissions annually [4, 5]. *Staphylococcus aureus* and viridans group streptococci are among the more common causative agents of IE, whereas group A *Streptococcus* (GAS) accounts for only 3% of cases [4]. GAS serotypes have been associated with severe invasive disease in other parts of the body, but there are only a few reports of IE caused by GAS [6]. Here, we report two cases of GAS endocarditis that were treated at our hospital in 2015 and 2016 and review the literature about GAS serotypes and IE.

### Case presentations

#### Patient 1

**Fig. 1:**
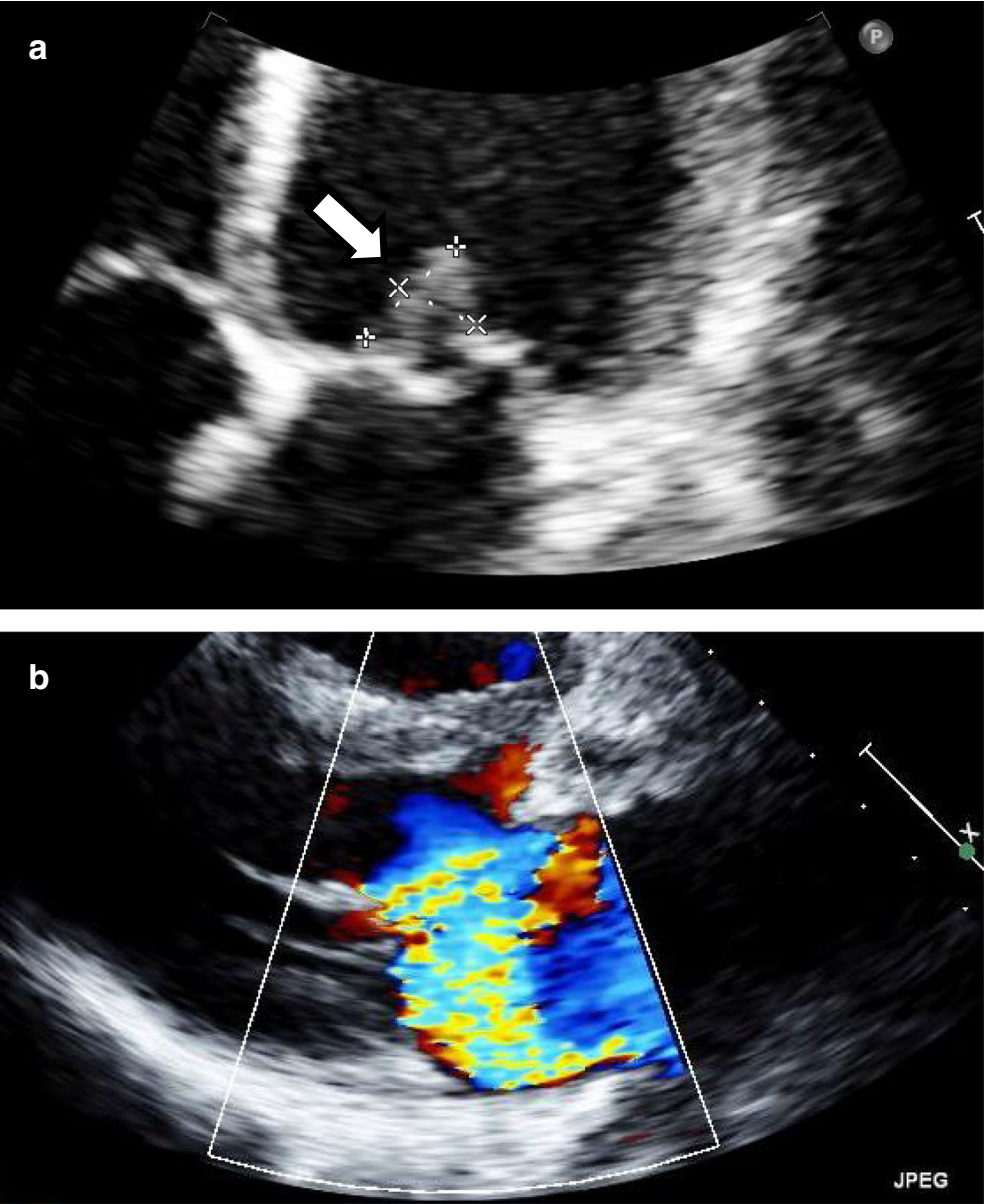
Echocardiogram. **a**. vegetation on mitral valve (arrow). **b.** moderate mitral regurgitation

**Fig. 2:**
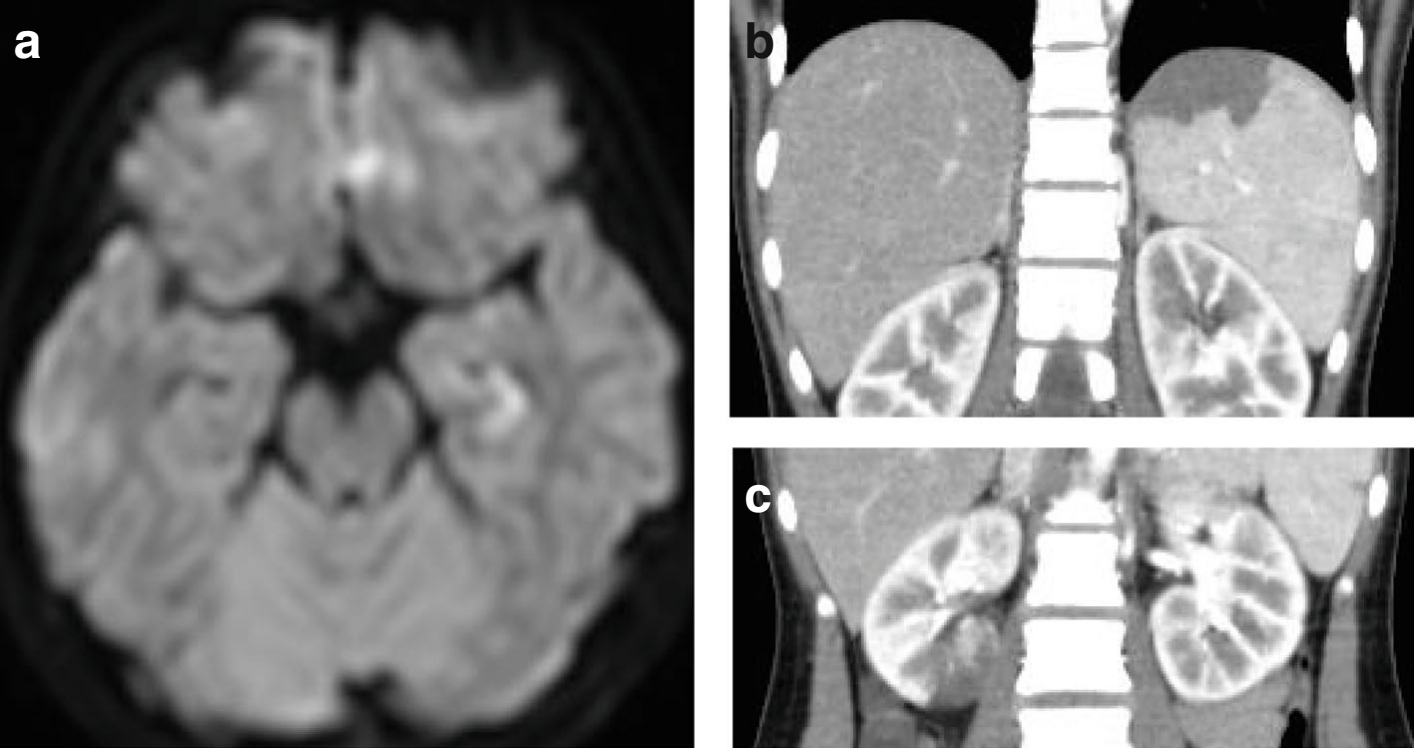
Imaging of infarction. **a**. Diffusion-weighted magnetic resonance imaging demonstrating infarction in deep left temporal cortex. **b**., **c**. Contrast enhanced computed tomography demonstrating infarction of spleen (**b**) and kidney infarction (**c**)

A previously healthy 14-year-old girl presented with 3 days of fatigue, 2 days of fever and behavior changes including becoming abnormally talkative, and 1 day of limp. She had black spots on her palms and soles, and black discoloration of the left second finger and left fifth toe. She came to our emergency department because of worsening pain in the left toe and because she was developing a confused mental status. Initial vital signs were: temperature 39.8 °C, heart rate 130 beats/min, blood pressure 100/71 mmHg, respiratory rate 18 breaths/min, and 98% oxygen saturation on room air. She was oriented but oddly garrulous. There were no hemorrhages in the palpebral conjunctiva but the uvula and posterior pharynx were covered with petechial hemorrhages suggesting streptococcal pharyngitis. No cardiac murmurs were auscultated. Her left second finger and left fifth toe were black, she had petechiae on the right palm, and the dorsum of the left foot was erythematous, warm, and swollen. Initial laboratory evaluation showed signs of disseminated intravascular coagulation (DIC) with an elevated white blood cell count and an elevated C-reactive protein. We thought that the skin and soft tissue infection of the finger and toe were causing bacteremia and DIC. GAS was suspected as the causative microorganism, and she was started on intravenous (IV) ampicillin/sulbactam and clindamycin. Two of three blood cultures grew *Streptococcus pyogenes* (T6 M6, *emm*6.104). Based on susceptibility testing results, the ampicillin/sulbactam was switched to just ampicillin and she was continued on clindamycin. Her mental status had almost cleared by the end of the first hospital day and she was wholly awake and alert by hospital day 3. A new systolic ejection murmur was heard, and echocardiography showed vegetation on the mitral valve with mitral regurgitation (MR) (Fig. 1). She also had findings of infarctions in the deep left temporal cortex, spleen, and kidney (Fig. 2). Two weeks later, she developed an erythematous rash over her entire body. A drug rash was suspected and she was switched from ampicillin and clindamycin to cefotaxime. There was no sign of heart failure, however, the MR worsened. She was started on a diuretic and an angiotensin-converting enzyme inhibitor. The left fifth toe became necrotic and was amputated on hospital day 45. Subsequently, she remained stable and was discharged. Two months later, she underwent mitral valve repair. She has remained on angiotensin-converting enzyme inhibitor therapy, and one year later, shows no signs of neurological sequelae or heart failure.

#### Patient 2

**Fig. 3:**
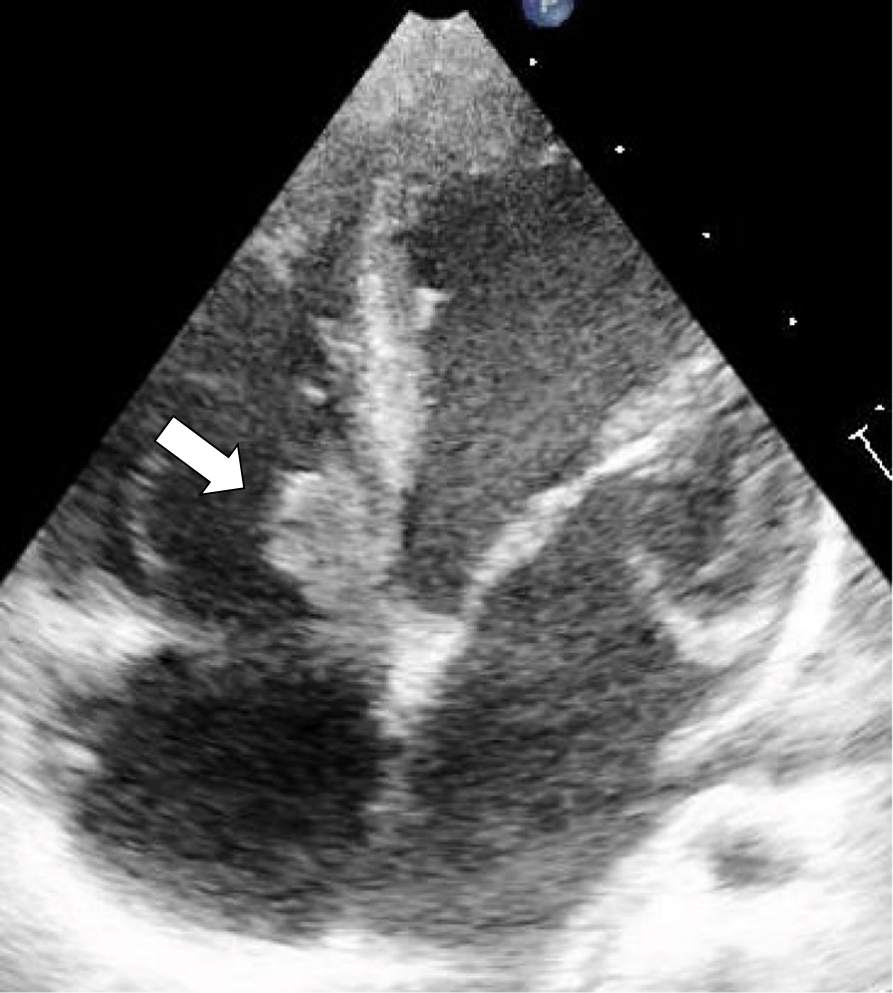
Apical four-chamber view. Vegetation on membranous septal aneurysm (arrow)

A 17-month old boy presented to a neighboring municipal hospital secondary to fever. He had a past history of spontaneous closure of a ventricular septal defect (VSD) at 5 months of age with residual mild MR. He presented with a 2-day history of fever and a 1-day history of irritability. He was referred to our hospital because of elevated white blood cell count and elevated C-reactive protein. Initial vital signs were: temperature 40.6 °C, systolic blood pressure 100 mmHg, heart rate 157 beats/min, respiratory rate 60 breaths/min, and 97% oxygen saturation in room air. He had no conjunctival petechiae, no splinter hemorrhages of the nails, and no rash. No heart murmurs were auscultated. Initial laboratory evaluation was significant for a white blood cell count of 14,300 cells/ml and a C-reactive protein level of 30.89 mg/dL. Blood cultures were drawn at both the municipal hospital and our hospital 12 h apart, and he was empirically started on IV ampicillin and cefotaxime for possible bacteremia. Two blood cultures grew *S. pyogenes* (T4 M4, *emm*4.0). On hospital day 5, echocardiography showed vegetation on a right-sided membranous septal aneurysm (MSA) (Fig. 3) and he was diagnosed with IE. Based on susceptibility testing results, the cefotaxime was stopped and ampicillin only was continued. While receiving antibiotic therapy, he did not develop any complications such as pulmonary hypertension or pulmonary embolism. IV ampicillin was continued for 4 additional weeks after follow-up blood cultures were determined to be negative, and he was ultimately discharged. The patient has shown no signs of heart failure during the 2-year follow-up period.

## Discussion and conclusions

GAS, also known as *S. pyogenes*, is best known for causing mild infections such as pharyngitis and impetigo, but it can also cause serious infections such as bacteremia, cellulitis, necrotizing fasciitis, and streptococcal toxic shock syndrome [6]. Invasive GAS (iGAS) disease is defined as the entry of GAS into usually sterile sites of the body [6]. The morbidity and mortality of iGAS infection can be high, and in 2005 the World Health Organization reported 663,000 new iGAS cases with 163,000 iGAS-related deaths that year [7]. Risk factors for iGAS diseases among adults include human immunodeficiency virus infection, cancer, heart disease, diabetes, lung disease, alcohol abuse, injection drug use, and pregnancy- related factors. Among children, varicella is a prominent risk factor [8]. Nelson et al. reported that from 2005 to 2012, in patients of all ages with iGAS infections, frequencies of bacteremia, skin and soft tissue infections, and endocarditis or pericarditis in the United States were 24.9, 40.7, and 1.4%, respectively [9]. Additionally, Plainvert et al. reported that between 2006 and 2010, in patients aged ≥18 years in France, rates of bacteremia, skin and soft tissue infections, and endocarditis were 25.4, 43.7, and 0.6%, respectively [10].

Host genetic factors, such as human fibrinolytic protease plasmin, and various invasive factors including M protein, have been documented as contributing to iGAS diseases [11]. M protein is a major surface protein and a critical virulence determinant, and plays a crucial role in adherence and resistance to opsonophagocytosis [6]. Historically, GAS isolates were typed using serotype-specific antiserum raised against M protein. GAS strains are now more commonly categorized based on the *emm* gene encoding M protein [6]. A remarkable difference in the geographical distribution of *emm* types has been reported, and the isolation frequency for *emm* types from different GAS diseases parallels their rate of asymptomatic carriage in the same population [12, 13]. Moreover, there are significant associations between some *emm* types and disease severity. Examples include the association of *emm* types 2, 4, 6, and 12 with superficial disease and *emm* types 1 and 3 with invasive disease [14]. Little is known, however, about the relation between IE and GAS *emm* types. In several case reports, *emm* types 1 and 3 were most commonly linked to invasive disease, but M protein nontypeable strains can also cause iGAS diseases [15–17].

The M protein mainly plays three roles in iGAS disease: 1) adherence to the host cell, 2) resistance to host immune defense systems, and 3) gene regulation in response to environmental stress conditions [1, 6]. Initial bacterial attachment is hypothesized to be a two-stage process, first involving lipoteichoic acid and surface proteins such as pili, followed by more specific, high-affinity binding including M proteins [6]. M6 protein binds directly to ligands present on host cells [6]. Additionally, M1, M3, and M6 proteins may promote bacterial colonization by binding directly to components of the extracellular matrix [4, 6]. In resistance to host immune defense systems, M protein plays a crucial role in resistance to opsonophagocytosis. Streptococcal inhibitors of complement (SIC) produced by M1 and M57 strains also inhibit the binding of C5b67 complexes to cell membranes [18]. Fibronectin-binding protein FbaA is encoded in the genome of GAS serotypes 1, 2, 4, 9, 13, 22, 28, 44, 49, 60, 67, 75, 77, 79, 80, 82, 87, and 89, and it inhibits C3 deposition on bacterial cells to promote survival in human blood [19, 20]. Control of the two-component regulatory system *covRS* is required for GAS survival under general environmental stress conditions. Spontaneous and unidirectional mutations within *covRS* affect the expression of numerous virulence factors important for the initiation and progression of iGAS disease. The frequency of *covRS* mutations in non-M1 T1 serotypes is lower than that in the M1 T1 strain, which may explain why non-M1 T1 serotypes are less frequently isolated from human invasive infections [6, 11].

GAS is considered to be one of the less common etiologic organisms of IE. In pediatric IE, *S. aureus*, viridans group streptococci, coagulase-negative staphylococci, and GAS comprise 57, 20, 14, and 3% of all cases, respectively [4]. Additionally, IE occurs less often in children than in adults, accounting for approximately 1 in every 1300–2000 pediatric admissions annually [4]. Risk factors include congenital heart diseases (CHD) and central indwelling venous catheters [3]. Risk factors for mortality among patients without preexisting heart disease include premature/neonatal age and *S. aureus* as an etiologic agent [4]. Our patients had *emm* types 6 and 4, both generally considered to be associated with more superficial diseases. Patient 1 (Case M in Table 1) was previously healthy with no known risk factors. She was found to have T6 M6 proteins. Patient 2 (Case N in Table 1) had a history of spontaneous closure of a ventricular septal defect and he was found to have T4 M4 proteins. Subsequent to the 2007 AHA guidelines for prevention of IE, patients with native unrepaired cardiac lesions such as VSD have been considered to be at low risk for IE [21].

**Table 1:** *Streptococcus pyogenes* endocarditis and serotypes/*emm* types

Case [ref]	Year	Age, Sex	Underlying conditions	T type	M type	*emm* type*
A [15]	1992	3 y, Female	Arnold-Chiari malformation, ventriculoperitoneal shunt, developmental delay	12		
B [28]	2000	2 y 9 m, Male		12	nontypeable	
C [29]	2013	60 y, Male	smoking, nonalcoholic steatohepatitis, left iliofemoral bypass			**44**
D [16]	2015	63 y, Male				**12**
E [17]	2015	9 y, Female	VSD^a^ repair at 7 y	13		90.2
F [30]	2001	73 y, Female	Predisposing condition for IE			77
G [30]	2005	64 y, Male				82
H [30]	2006	33 y, Male	Intravenous drug user			82
I [30]	2008	68 y, Female				87
J [30]	2011	24 y, Male	Intravenous drug user			66
K [30]	2013	39 y, Male	Intravenous drug user, Prosthetic valve			22
L [30]	2013	51 y, Male	Intravenous drug user			75
M [Patient 1]	2016	14 y, Female		6	6	**6.104**
N [Patient 2]	2015	1y 5 m, Male	VSD^a^ (spontaneous closure at 5 m)	4	4	**4**

^a^*VSD* ventricle septal defect. ^*^emm types related to superficial disease are noted in bold font

### Review of the literature

We searched PubMed and J-STAGE databases by using search terms “infective endocarditis and group A streptococcus or *Streptococcus pyogenes*”. We found twelve case reports that contained detailed information and serotypes or *emm* types (Table 1) [22].

None of the strains isolated in these seven cases had M1 protein which is associated with a higher frequency of *covRS* mutations than the non-M1 T1 strains. These serotypes are less commonly associated with iGAS diseases. M6 (Case M, Patient 1) is known to more readily adhere to host cells, and M4 (Case N, Patient 2) inhibits complement deposition, which may have contributed to the development of IE. T12 (Cases A and B) is found in more than 13 *emm* types, thus, it is difficult to discuss invasiveness according to T types [23]. Case D was noted to have *emm*12 which is generally associated with mild infection, but it has also been significantly found in nephritis-associated GAS serotypes including M1, M55 (Case L), and M57. These *emm* types produce highly antigenic secretory proteins called SIC [6]. In case C, *emm* 44 was noted, which is also associated with mild infection. As previously noted, M44 strains produce FbaA, which inhibits complement deposition [6]. Emm serotypes of Cases F, G, H, I, and K are also known to produce FbaA. Furthermore, Oppegard et al. reported to detect FbaA in Case J. In case E, *emm*90 was noted. Fiona et al. described two cases of GAS necrotizing fasciitis caused by *emm*90 strains from 1990 to 1998 in Australia [24]. Guliz et al. reported 1428 isolates from Hawaii between 2000 and 2005 representing 21 different *emm* types that were never reported in the continental United States during or before surveillance studies, including *emm*90 strains [25]. Additionally, Chen et al. identified 20 cases of iGAS diseases caused by *emm*90 between 2005 to 2007 in Hawaii [26]. They described that *emm*90 strains appeared to be common after 2006 among iGAS disease-causing isolates. Therefore, with this many cases of iGAS being caused by *emm*90 strains, the mechanism by which *emm*90 strains cause invasive disease should be further elucidated.

Some categories of preexisting heart disease serve as risk factors for IE. Congenital heart diseases account for 80% of these cases, while previous rheumatic heart disease, prosthetic valves, and cardiomyopathies account for the majority of the rest [4]. Tetralogy of Fallot is the most common congenital heart disease associated with IE (19.8%), followed by VSD (18%) [4]. Corrective surgery for children with VSD, atrial septal defects, or patent ductus arteriosus with no residual defect is said to eliminate the attributable risk for endocarditis 6 months after surgery [3]. Cases E and N should therefore be considered not to have had added risk factors for developing IE since their VSD’s had either been surgically corrected or had resolved spontaneously. Although the 2007 AHA antibiotic prophylaxis guidelines for the prevention of IE no longer consider native unrepaired cardiac lesions to be a high or moderate risk for IE, IE is known to occur in these patients [21]. Knirsch et al. contend that IE should be considered a lifetime risk for non-operated, repaired, and palliated CHD, including VSD [27]. We suspect that the GAS capsule, which inhibits complement deposition, quite possibly played a role in the development of IE in Patient 2.

In conclusion, GAS is a rare etiologic organism of IE, and there are few reports of GAS endocarditis with specific information on serotypes/*emm* types. In general, most serotypes/*emm* types associated with GAS typically cause only mild infection, but as this case report shows, GAS can be associated with serious invasive disease such as IE. A greater accumulation of cases is necessary to elucidate the association between serotypes/*emm* types and GAS IE.

## Abbreviations

CHD: Congenital heart disease
DIC: Disseminated intravascular coagulation
GAS: Group A streptococcus
IE: Infective endocarditis
iGAS: Invasive GAS
IV: intravenous
MR: Mitral regurgitation
MSA: Membranous septal aneurysm
SIC: Streptococcal inhibitor of complement
VSD: Ventricle septal defect
